# Artificial Intelligence in Coronary Computed Tomography Angiography: A Bibliometric Analysis of Trends and Themes

**DOI:** 10.1002/clc.70447

**Published:** 2026-08-24

**Authors:** Shanshan Jiang, Yucong Zheng, Keqin Pan, Lu Ma

**Affiliations:** ^1^ Department of Radiology Tsinghua University Hospital Beijing China

**Keywords:** artificial intelligence, bibliometric analysis, CiteSpace, computed tomography angiography, coronary artery disease, VOSviewer

## Abstract

**Background:**

Artificial intelligence (AI) in coronary computed tomography angiography (CCTA) is increasingly recognized, but comprehensive bibliometric analyses on its integration are scarce.

**Hypothesis:**

This study aims to bridge this gap by delineating research trajectories, influential entities, and evolving themes within this field.

**Methods:**

A bibliometric analysis was conducted on literature from the Web of Science Core Collection, spanning publications from January 2007 to October 2024. Analysis tools included VOSviewer, CiteSpace, and R‐bibliometrix, which were used to map and visualize the publication trends, collaborative networks, and thematic evolution. A manual relevance audit and a sensitivity analysis using a narrower query were performed to validate the search strategy.

**Results:**

A total of 499 articles were analyzed. The number of publications increased annually in the past decade, with a peak of 113 in 2022. China led with 193 articles, and the USA served as a central node in the cooperation network. The Medical University of South Carolina and Yonsei University tied for first with 54 articles each. Schoepf U. Joseph was the most influential author. *European Radiology* led in h‐index and total publications. The keyword analysis revealed “coronary artery disease” as the most frequent, followed by “deep learning” and“machine learning.” Notable burst keywords included “fractional flow reserve,” “diagnostic accuracy,” and “SCCT guidelines.”

**Conclusion:**

The field of AI in CCTA is rapidly evolving, with increasing international collaboration and a focus on technological advancements. Machine learning prediction has emerged as a bibliometrically prominent theme with intense citation activity, warranting further validation and standardized integration into clinical practice.

## Graphical abstract

AI in CCTA research is rapidly expanding, led by China and the USA, with deep learning and machine learning as central themes. Key areas like fractional flow reserve and diagnostic accuracy show intense recent interest, highlighting a shift toward predictive applications requiring further clinical validation.

**Abbreviations**
AI: artificial intelligence
ASPC: American Society for Preventive Cardiology
CAD: coronary artery disease
CCTA: coronary computed tomography angiography
FFR: fractional flow reserve
IF: impact factors
JCR: Journal Citation Reports
WoSCC: Web of Science Core Collection

## 1 Introduction

Coronary CT angiography (CCTA) is a non‐invasive cardiac imaging technique that leverages X‐ray computed tomography (CT) to produce detailed, three‐dimensional images of the coronary arteries, facilitating the non‐invasive assessment of coronary artery disease (CAD) [1]. CCTA has found extensive applications in diagnosing various cardiovascular conditions, including coronary artery stenosis, atherosclerosis, and plaque formation [2]. Its advantages lie in the high spatial and temporal resolution, which allows for the accurate detection of anatomical abnormalities and functional impairments within the coronary arteries. Furthermore, CCTA offers a cost‐effective and relatively low‐risk alternative to invasive diagnostic procedures such as conventional angiography [3]. However, traditional CCTA analysis is labor‐intensive, time‐consuming, and demands considerable expertise and skill from clinicians [4, 5]. With the emergence of artificial intelligence (AI), researchers have begun to explore its applications in CCTA to enable automated and intelligent diagnosis and analysis [6].

AI, a transformative 21st‐century technology, has advanced rapidly across fields like machine learning (ML), deep learning, and computer vision, impacting human life in unprecedented ways [7]. In healthcare, the application of AI is extensive and profound, encompassing various aspects such as diagnosis, treatment planning, and disease monitoring [8]. In radiology, AI models have shown high accuracy in imaging diagnoses of conditions like pulmonary nodules diagnosed by chest CT and other disorders [9]. Additionally, AI tools have been integrated into clinical treatment planning, including radiotherapy planning for breast cancer, improving the quality and efficiency of these plans [10]. Furthermore, AI systems demonstrate substantial potential in medication assistance, emergency triage, pathology diagnosis, cancer treatment, infectious disease research, and chronic disease management [11, 12]. These applications not only improve diagnostic and treatment accuracy but also enhance the efficiency and accessibility of healthcare systems [10].

Currently, AI has been applied in various aspects within the field of CCTA. In automated quantitative analysis, deep learning models can quantify the volume, composition, and distribution of coronary plaques, thus enhancing diagnostic accuracy and consistency [13]. Functional assessment utilizes AI techniques to calculate fractional flow reserve (FFR), such as CT‐FFR, which aids in evaluating the functional significance of coronary stenosis [14]. For risk prediction, AI models combine CCTA images with clinical data to forecast cardiovascular event risks, such as myocardial infarction [15, 16]. Additionally, AI algorithms improve CCTA image quality by reducing noise and artifacts, thereby increasing diagnostic reliability [17]. Despite significant advancements, AI applications in CCTA still face challenges. Its accuracy and reliability are limited by data quality and algorithm complexity [13, 18]. Additionally, training AI models requires large amounts of labeled data, which is costly and time‐consuming [19]. Variations in medical devices and scanning protocols may also affect the generalizability of AI algorithms [20]. To further address these issues, a systematic and comprehensive understanding of the current trends and themes in AI‐based CCTA research is essential. This necessitates a rigorous analysis of the published literature, identifying key research areas, emerging technologies, and potential directions in the future.

Bibliometric analysis utilizes mathematical and statistical methods to examine all articles published on a specific research topic over a defined period, offering insights into research categories, co‐authorship networks, keyword frequency, and the most cited articles or journals [21, 22]. Previous literature has conducted bibliometric studies on the applications of AI in CT [23], as well as the most‐cited original articles in cardiac CT [24]. However, there is a lack of bibliometric studies specifically focused on the application of AI in CCTA. Therefore, this study aimed to conduct a bibliometric analysis of the trends and themes in AI‐based CCTA research, to provide a comprehensive overview of the current state of the field, identify emerging trends, and highlight potential areas for future research.

## 2 Materials and Methods

### 2.1 Search Strategy and Data Collection

A comprehensive literature search was performed using the Web of Science Core Collection (WoSCC), covering the Social Sciences Citation Index (SSCI) and the Science Citation Index Expanded (SCIE). The search was tailored to identify studies combining “Coronary CT angiography” with “Artificial Intelligence,” using the following search string: ((TS = (“CT angiography” OR “computed tomograph* angiography” OR CTA OR CCTA)) AND TS = (coronary OR cardiac)) AND TS = (“artificial intelligence” OR AI OR “deep learning” OR “robotic*“ OR “supervised learning” OR “unsupervised Learning” OR “computer vision system” OR “computational intelligence” OR “machine learning” OR “evolutionary computation” OR “ensemble learning” OR “reinforcement learning” OR “neural network” OR “data learning” OR “expert systems” OR “fuzzy logic” OR “natural language processing”). The final search was performed on October 15, 2024. Only English‐language publications were included, as the field's primary output is in English and bibliometric tools have limited compatibility with non‐English records. Document types were restricted to Articles, Reviews, and Early Access; Editorials, Letters, Meeting Abstracts, Book Chapters, Corrections, Data Papers, and Withdrawn publications were excluded due to insufficient peer‐reviewed research depth. Data on publication years, countries/regions, institutions, author details, and keywords were extracted in “Full record and cited references” and “plain text” formats for detailed bibliometric assessment.

Eligibility was determined using the following criteria: (i) studies involving CCTA, CT‐derived FFR (CT‐FFR), or coronary artery calcium scoring on coronary CT; (ii) application of AI, ML, deep learning, neural networks, computer vision, or radiomics with ML; (iii) outcomes related to image analysis, diagnosis, risk prediction, functional assessment, image reconstruction, plaque quantification, or workflow optimization. Studies were excluded if they focused on non‐CCTA (e.g., TAVI‐CT, aortic CTA), non‐AI approaches (e.g., traditional iterative reconstruction without deep learning), non‐CCTA imaging modalities (e.g., cardiac MRI, IVUS, OCT), or non‐imaging AI applications (e.g., ECG‐based deep learning, facial‐photo analysis).

### 2.2 Data Cleaning and Disambiguation

Deduplication was performed using bibliometrix::duplicatedMatching() in R, with a title similarity threshold of 0.95, DOI matching, and first‐author verification. Author name variants (e.g., “Schoepf U. Joseph”/“Schoepf UJ”) were harmonized using a VOSviewer thesaurus file through manual review. Institution names were standardized based on the WoS Organization‐Enhanced field; variant entries for the same entity (e.g., “Siemens AG”/“Siemens Healthcare”/“Siemens Medical Solutions”) were merged, as were university‐hospital pairs (e.g., “Yonsei University” and “Yonsei University Health System”). Keyword synonyms were consolidated via a thesaurus file prior to analysis: multiple variants of CCTA (e.g., “ct angiography,” “computed‐tomography angiography,” “computed tomographic angiography”) were merged into “coronary CT angiography”; “coronary‐artery‐disease” and “artery‐disease” were unified as “coronary artery disease”; abbreviations such as “ffr” were mapped to “fractional flow reserve”; singular/plural and hyphenation variants (e.g., “stenoses”/“stenosis,” “accuracy”/“diagnostic‐accuracy”) were merged accordingly. Non‐topical descriptors (e.g., “expert consensus document,” “society,” “multicenter”) and overly generic terms (e.g., “disease,” “management”) were removed.

### 2.3 Statistical Analysis and Visualization

For our statistical analysis, we employed VOSviewer (version 1.6.20) and CiteSpace (version 6.3.R1), alongside R‐bibliometrix (version 4.3.3). VOSviewer was crucial for mapping co‐authorship and institutional collaboration networks. Additionally, co‐occurrence analysis of keywords was constructed to reveal research hotspots. The software visualized the strength and frequency of links between nodes, with node size indicating the frequency of occurrence in titles and abstracts, providing a graphical representation of collaboration intensities. CiteSpace was configured for keyword citation bursts analysis to identify the primary focus within our dataset. We set time slicing from January 2007 to October 2024, focusing on keywords as the node type. Each slice's top 5 were extracted, with adjustments made through pathfinder and pruning merged networks to clarify and enhance the visual outputs. Burst detection used Kleinberg's algorithm with *γ* = 1.0. This method produced a dynamic timeline visualization of keyword evolution, highlighting pivotal developments in the application of AI in CCTA.

Through using R‐bibliometrix, we conducted further analyses to ascertain the h‐index, g‐index, and m‐index, assessing the academic impact across the collected publications and journals. These metrics helped quantify the influence and scholarly reach of the research outputs [25, 26]. Journals were also evaluated based on their Journal Citation Reports (JCR) 2023 impact factors (IF) and 2023 quartile ranking (Q1−Q4), providing a context for the prestige and relevance of the publications within the field. All bibliometric analyses employed full counting. Author names were drawn from the AU field, institutional affiliations from the C1 field, and keyword analyses combined Author Keywords (DE) with Keywords Plus (ID). In VOSviewer, the minimum publication threshold was set at 4 for both author and institution co‐authorship networks, and the minimum co‐occurrence frequency for keywords was set at 3.

### 2.4 Search Strategy Validation

To assess dataset specificity, a manual relevance audit was performed on a random sample of 100 articles drawn from the broad query retrieval (set.seed = 20260422). Of these, 71 articles (71%) were confirmed as relevant and 29 (29%) were excluded. The primary exclusion reasons were: non‐coronary CTA modalities (cardiac MRI, TAVI‐CT, ECG‐based AI, IVOCT) in 14 cases, non‐coronary arterial CTA (aortic coarctation, internal mammary artery graft) in five cases, completely unrelated topics (surgery, stroke, veterinary medicine) in five cases, non‐AI studies in two cases, non‐coronary cardiac structures (left atrial appendage) in two cases, and CCTA used solely as a post‐surgical follow‐up tool in one case.

To further validate search sensitivity, a sensitivity analysis was conducted using a narrower query with the following search string: TS = ((“coronary CT angiography” OR “coronary computed tomography angiography” OR “CT coronary angiography” OR CCTA OR “CT‐FFR” OR FFRCT OR “fractional flow reserve CT”) AND (“deep learning” OR “machine learning” OR “artificial intelligence” OR radiomics OR “convolutional neural network* “OR” neural network* “OR” “computer vision” OR “automated segmentation” OR “automated quantification”)). This narrow query was executed on May 22, 2026, and therefore covered a wider time window than the original broad search; this was considered a conservative design, as concordance between the two strategies despite the expanded temporal coverage would further support the robustness of the original dataset. After applying identical document‐type filtering and language restriction, the narrow query yielded 698 records. Comparison between the broad query dataset (*n* = 499) and the narrow query dataset was performed across multiple dimensions using Jaccard indices and overlap rates, with results reported in the Sensitivity Analysis subsection of Results.

## 3 Results

### 3.1 The Publication and Citation Trends

**Figure 1:**
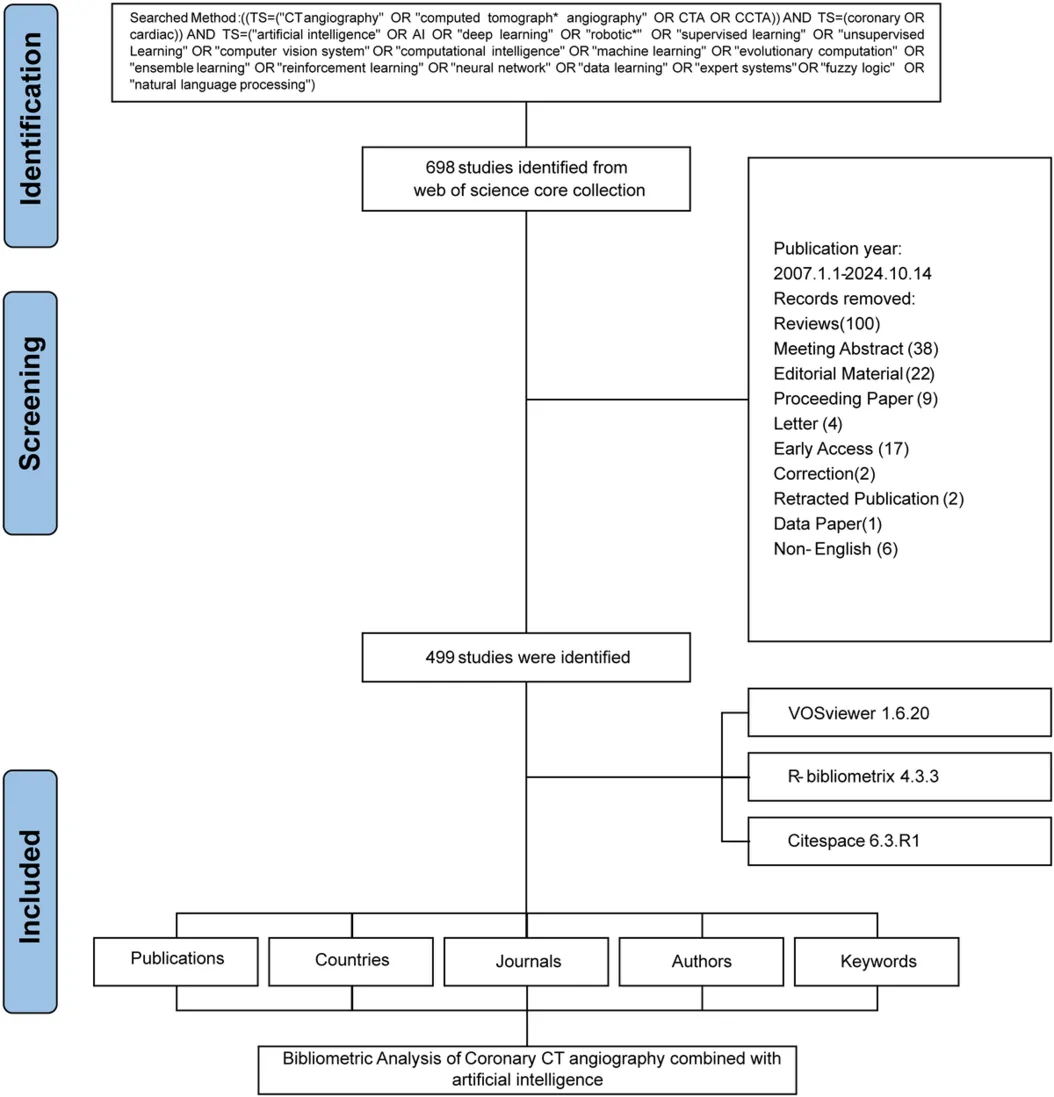
Flow diagram of the bibliographic retrieval process.

**Figure 2:**
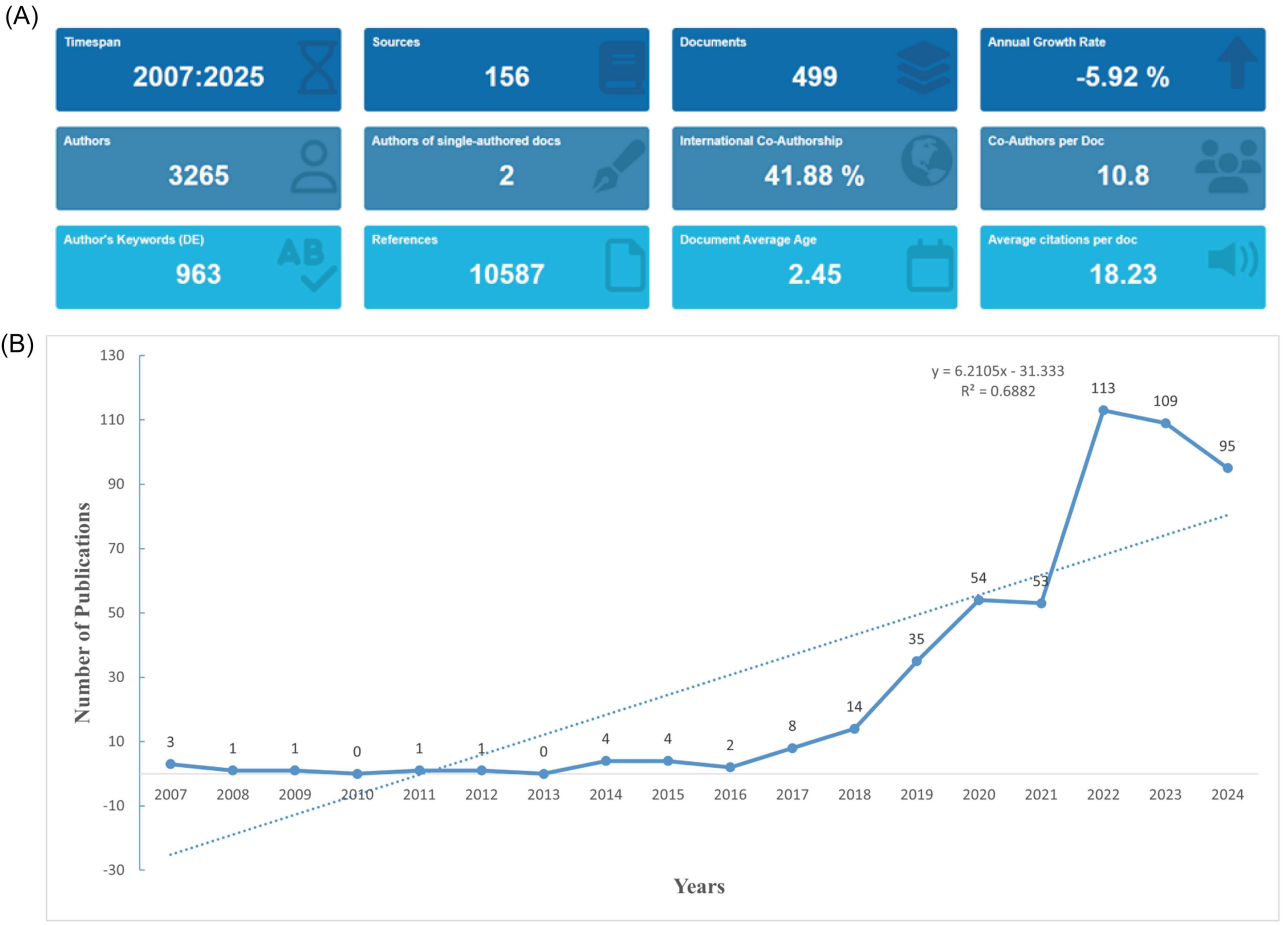
Analysis of the general information. (A) A summary of quantitative analysis of the publications. (B) Annual output of research from 2007 to 2024.

This strategy was designed to capture a broad spectrum of research articles written in English. The initial query retrieved 698 studies, narrowed down to 499 after excluding non‐relevant formats such as editorials, letters, meeting abstracts, book chapters, corrections, data papers, and withdrawn publications, followed by deduplication and manual relevance screening (Figure 1). The investigation showed that 3265 authors from 2422 institutions across 56 countries/regions contributed to these productions. These works were published in 156 journals, citing 10 587 references. The average citation age of documents is 2.45 years, indicating a reliance on recent literature. The average citation density of 18.23 citations per document indicates substantial scholarly attention, although this metric is influenced by the time‐dependent accumulation advantage of earlier publications (Figure 2A). The Annual Growth Rate (AGR), calculated as the mean yearly percentage change in publication count, may exhibit short‐term negative values during year‐to‐year fluctuations without contradicting the long‐term upward trajectory.

The annual publication output showed an overall upward trajectory from 2017 onward, despite year‐to‐year fluctuations (Figure 2B). The volume of articles in this field was relatively low in the early years (2007−2016), with a rapid increase since 2017 (8 articles), reaching a peak in 2022 (113 articles). The top 50 articles with the highest cited counts are exhibited in Supporting Information S1: Table 1.

### 3.2 Analysis of Leading Countries

**Figure 3:**
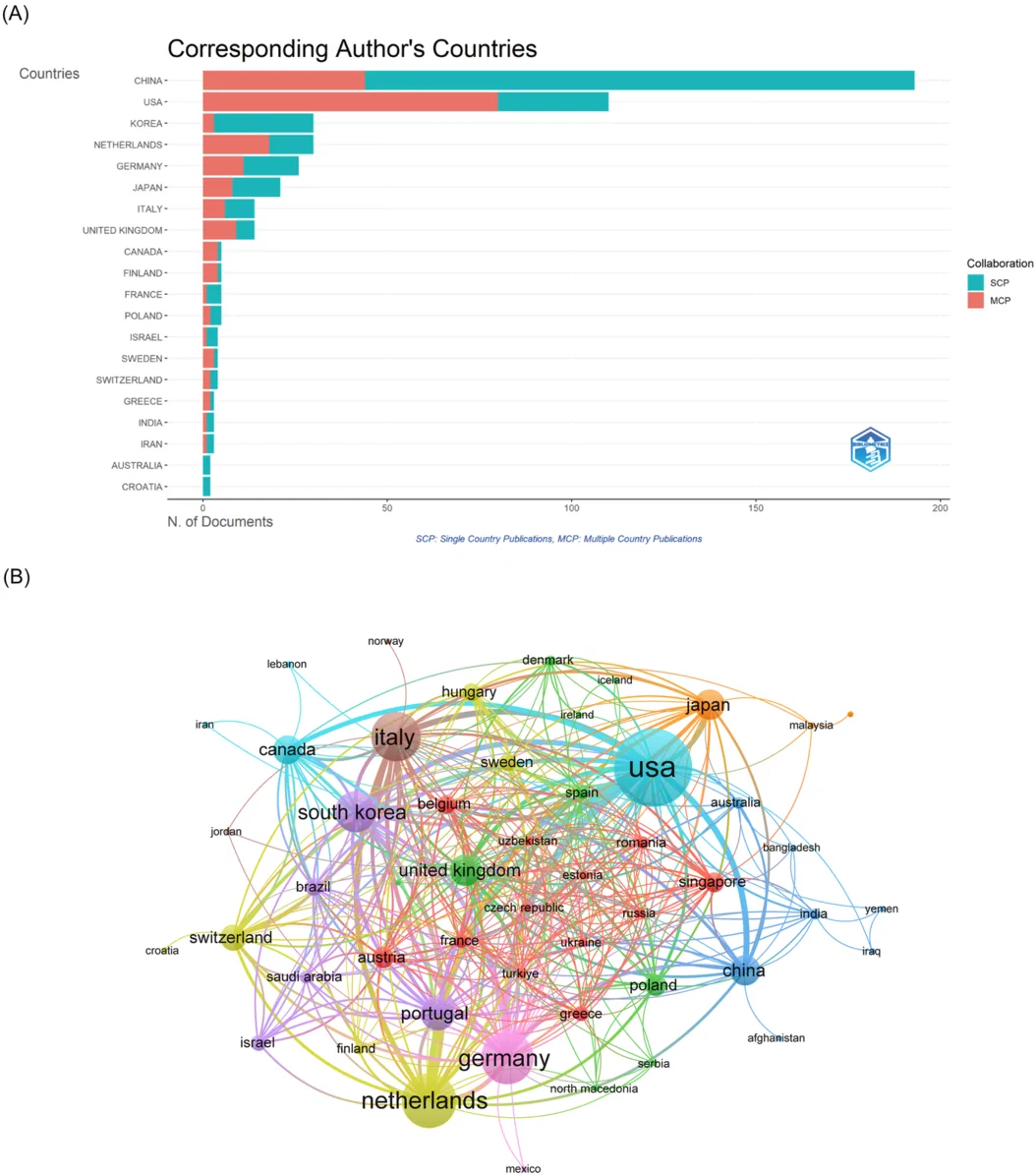
Analysis of countries. (A) Distribution of responding author's publications by country. (B) Visualization map depicting collaboration among different countries.

A total of 56 countries/regions were involved in these publications. China led with 193 documents (38.68% of the total), and ranked second in total citations (1791), with a Multiple Country Publications (MCP) ratio of 22.80%. The USA ranked second in number of articles (110, 22.04%) and first in total citations (3651), with a MCP ratio of 72.73%. Korea and the Netherlands respectively contributed 30 articles to this field (6.01%). Furthermore, the Netherlands and the United Kingdom also showed strong MCP ratios, 60.00% and 64.29%, respectively (Figure 3A, Supporting Information S1: Table 2).

The international collaboration network involving 56 countries or regions with a minimum of 1 article, positioned the USA (strength = 442) as a central node with strong ties to various countries, particularly in Europe such as the Netherlands (strength = 259, ranking second), Germany (strength = 245, ranking third), and Italy (strength = 225, ranking fourth). China (strength = 108, ranking eighth), though primarily focused on domestic collaborations, also maintains robust links with the USA, Japan, and Korea (Figure 3B).

### 3.3 Analysis of Leading Institutions

**Figure 4:**
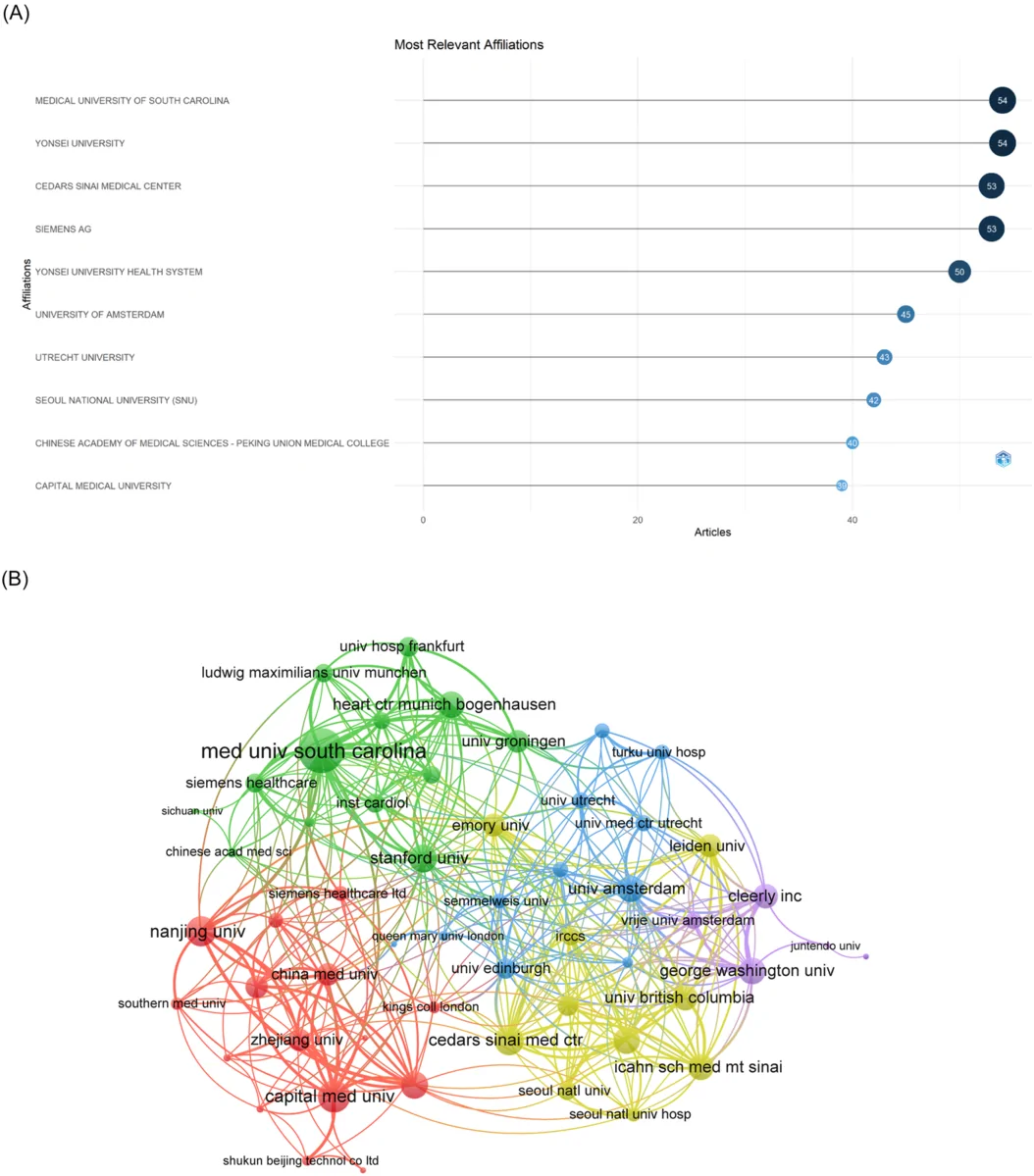
Analysis of institutions. (A) Top 10 institutions ranked by article count. (B) Visualization map depicting collaboration among different institutions.

2,422 institutions contributed to these publications. In terms of article counts, the Medical University of South Carolina in the USA and Yonsei University in Korea were tied for first place, each with 54 articles, followed by Cedars‐Sinai Medical Center in the USA (53 articles) and Siemens AG in Germany (53 articles) (Figure 4A).

Among the 114 institutions involved in international collaborations with a minimum of four articles, the Medical University of South Carolina emerged as a key hub with extensive connections to both national and international institutions (strength = 162). Other influential institutions, such as Capital Medical University (strength = 95) Cedars Sinai Medical Center (strength = 84), were also depicted, each forming strong collaborative clusters. These institutions connected with prominent research centers in the USA, China, fostering a dense network of knowledge exchange (Figure 4B).

### 3.4 Analysis of Authors and Co‐Authors

A total of 3265 authors have contributed to this research field, with high‐impact individuals highlighted in Supporting Information S1: Table 3. Schoepf U. Joseph ranked highest in terms of influence, with an H‐index of 16, 36 publications and 271 citations, marking him as a central figure in this area. Min James K., Chang Hyuk‐Jae and Pontone Gianluca followed closely, with H‐index of 15, 14, and 14, respectively. Tesche Christian ranked second in terms of citations (199).

**Figure 5:**
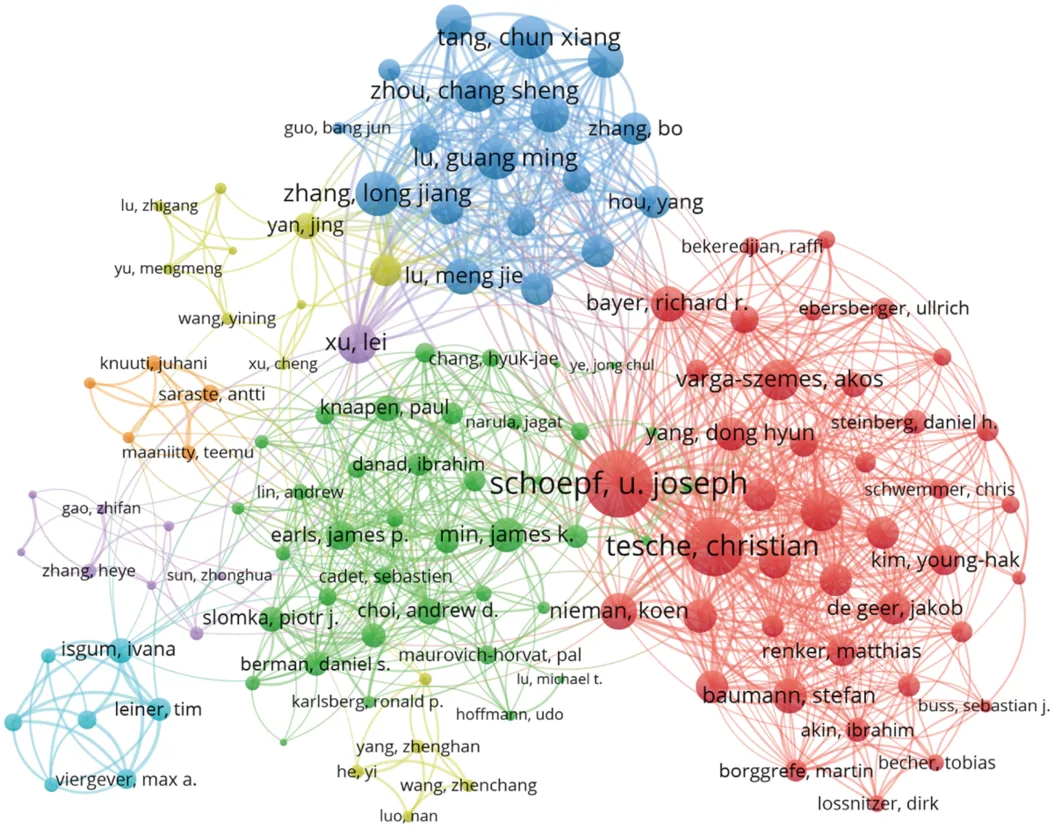
The visualization map depicting collaboration among authors.

The co‐authorship network illustrates the collaborative patterns among 168 authors with a minimum of collaborations of 4 articles (Figure 5). The network is divided into distinct clusters, each representing a major area of research focus. The red cluster, dominated by Schoepf U. Joseph (strength = 258) and Tesche Christian (strength = 209), shows a concentration in cardiovascular imaging, connecting strongly with other impactful authors such as Varga‐Szemes Akos and Yang Dong Hyun. Meanwhile, the blue cluster, led by Zhang Long Jiang (strength = 131) and Tang Chun Xiang (strength = 121), focuses on advanced imaging technology and is characterized by close intra‐group collaborations.

### 3.5 Analysis of Journals and Co‐Cited Journals

The collected publications cover a diverse range of research fields, with *European Radiology* (IF 2023 = 4.7, Q1) leading in H‐index (16) and total publications (35 documents). Other prominent journals include *JACC‐Cardiovascular Imaging* (H‐index = 12, IF 2023 = 12.8, Q1) and *Radiology* (H‐index = 9, IF 2023 = 12.1, Q1), both ranking highly in impact and collaboration within the field. Among the top 20 high‐impact journals, the *European Heart Journal* exhibited the highest IF 2023 of 37.6 (Supporting Information S1: Table 4).

**Figure 6:**
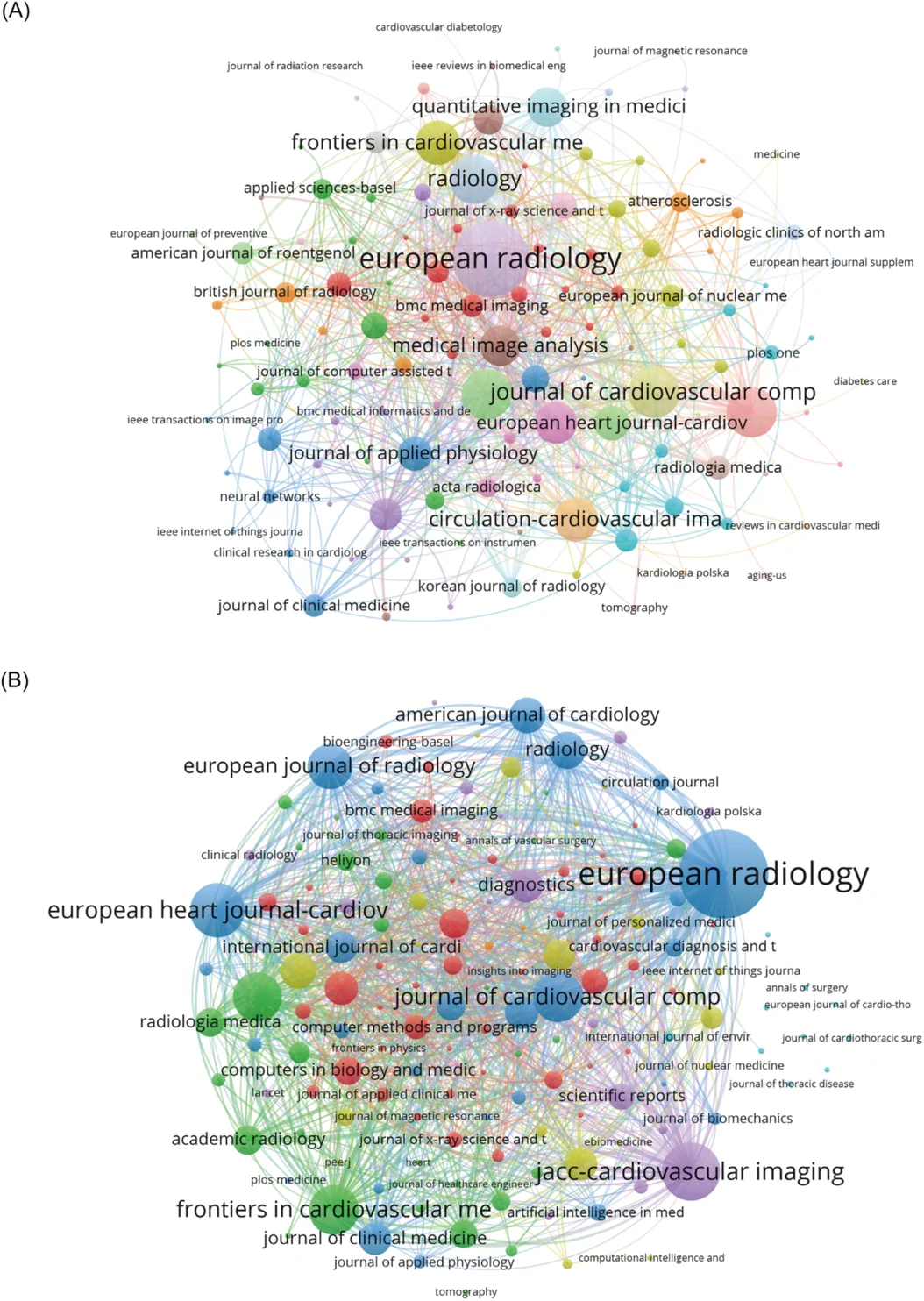
Analysis of journals. (A) The co‐occurrence networks of journals. (B) The coupling networks of journals.

The journal co‐occurrence network measures the frequency of joint citations among journals within the same articles, where strong links signify thematic convergence. Among 156 journals with at least 1 occurrence, the three key journals with the highest total link strength were *European Radiology* (strength = 220), *Journal Of Cardiovascular Computed Tomograph*y (strength = 115), *and JACC‐Cardiovascular Imaging* (strength = 112) (Figure 6A).

Journal coupling network evaluates interconnectedness by examining common references in articles, with strong links highlighting significant overlap in these references, thereby emphasizing a shared intellectual foundation. Among 156 journals with at least 1 coupling, the three key journals with the highest total link strength were the *European Radiology* (strength = 11,882), *JACC‐Cardiovascular Imaging* (strength = 5863), *and European Heart Journal‐Cardiovascular Imaging* (strength = 5236) (Figure 6B).

### 3.6 Analysis of Co‐Occurring Keywords and Burst Terms

**Figure 7:**
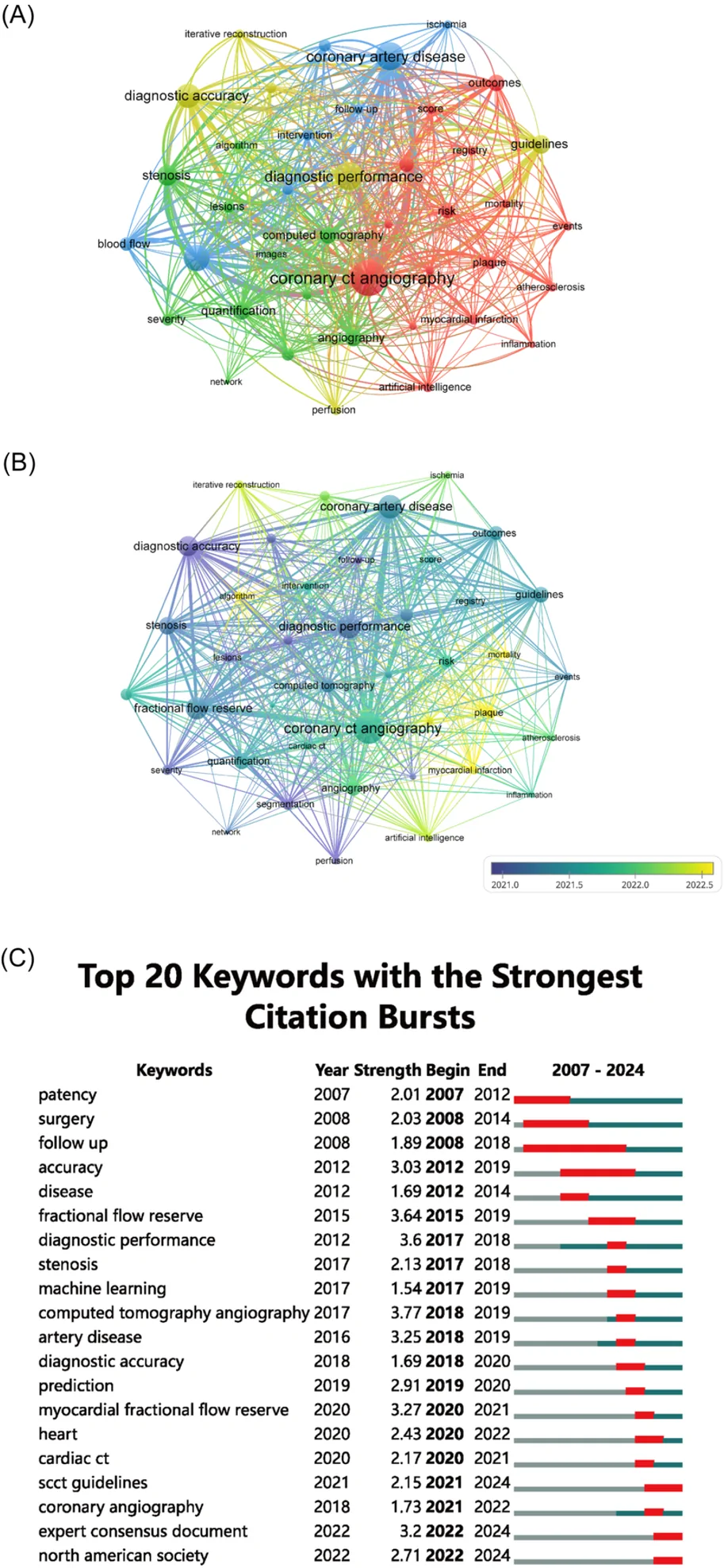
Analysis of keywords. (A) Visual analysis of keyword co‐occurrence network by different clusters. (B) Visual analysis of keyword co‐occurrence network in accordance with the appearance for the average time. (C) Top 20 keywords with the strongest citation bursts.

The analysis of keywords provides a comprehensive view of research hotspots and emerging trends within the field. A total of 111 keywords with a minimum of 3 occurrences were identified and grouped into 11 clusters, indicated by different colors (Figure 7A). The keyword with the highest frequency is “coronary artery disease” (139 occurrences) belonging to the blue cluster, which includes keywords related to cardiovascular diseases and diagnosis, such as “atherosclerosis” and “fractional flow reserve”. The second most frequent keyword is “deep learning” (107 occurrences), belonging to the red cluster, which is related to advanced imaging techniques and AI, including “convolutional neural network” and “image segmentation”. The third most frequent keyword is “machine learning” (89 occurrences), belonging to the orange cluster, which is associated with data‐driven medicine and imaging, also encompassing “radiomics” and “cardiac computed tomography.” Also noteworthy is the purple cluster, which pertains to imaging techniques and cardiovascular pathology, encompassing keywords like “computed tomography angiography,” “coronary stenosis,” “atherosclerotic plaque,” and “radiation dose.”

Furthermore, these keywords have been further classified based on their average occurrence time (Figure 7B). This visualization reveals that early studies (dark green) focused on foundational topics, such as “coronary artery disease,” “machine learning.” More recent research, represented by light green, has shifted towards technological advancements, with keywords like “deep learning,” and “radiomics.” The latest keywords, such as “transcatheter aortic valve implantation,” “digital health,” “motion correction,” “U‐net,” representing emerging technologies.

Figure 7C highlights the top 20 keywords with the strongest citation bursts, offering insight into shifting research priorities over time. Before 2010, the keyword “follow up” (strength = 1.89) experienced the longest burst period from 2008 to 2018. Between 2010 and 2020, notable citation bursts include “fractional flow reserve” (strength = 3.64), “machine learning” (strength = 1.54), “diagnostic accuracy” (strength = 1.69), and “prediction” (strength = 2.91). Since 2021, keywords like “SCCT guidelines” (strength = 2.15) and “expert consensus documents” (strength = 3.2) have shown strong bursts.

### 3.7 Sensitivity Analysis

To evaluate the robustness of the broad search strategy, a sensitivity analysis was conducted using a narrower query restricted to core CCTA/CT‐FFR and AI terminology. After applying identical document‐type and language filters, the narrow query retrieved 698 records. Comparative analysis across four available dimensions revealed high concordance between the two datasets. The Jaccard index for the top 10 contributing countries was 0.82 (9 of 11 unique countries shared), exceeding the predefined threshold of 0.80 and indicating highly overlapping geographic structure. The top 10 most‐cited articles showed an overlap rate of 80% (8 of 10 shared), meeting the threshold of 8/10 and confirming a consistent core knowledge base. Only one article in the narrow query's top 10 highly cited list (Chan K, 2024, *Lancet*) was not covered by the broad query's top 50, falling well below the threshold of 3 articles and representing a recently published precision‐targeted study. The Jaccard indices for the top 10 authors and top 10 journals were both 0.67 (8 of 12 unique entries shared); although these fell below the 0.80 threshold, the core 8 authors and 8 journals were identical between the two queries, and the peripheral ranking differences were attributable to the expanded temporal coverage and minor variations in retrieval scope rather than systematic divergence. Taken together, these findings indicate that the broad search strategy captured the core bibliometric structure of AI in CCTA research, and that the combination of broad retrieval with manual screening ensured both adequate sensitivity and acceptable specificity (the detailed manual audit verdict is provided in Supporting Information S1: Table 5; Supporting Information S1: Table 6).

## 4 Discussion

This bibliometric analysis examines global research trends and hotspots in AI applications within CCTA. The number of publications in this field has grown rapidly in the past decade due to the rapid development of AI. Our study identified key thematic areas, leading contributors, and emerging trends, highlighting a shift towards AI‐driven techniques in CCTA over recent years.

China and the USA emerged as the leading contributors, with distinct collaboration patterns reflecting each country's research focus and network reach. The USA's extensive international collaboration ratio underscores its role in connecting global research efforts, while China's predominantly domestic focus demonstrates its independent advancements in this domain. The institutional analysis identified key research hubs, such as the Medical University of South Carolina, Yonsei University, and Cedars Sinai Medical Center, each contributing significantly to the literature. These institutions, along with those in China and Korea, form dense collaborative networks, offering essential resources and expertise. Journals like *European Radiology* and *JACC‐Cardiovascular Imaging* serve as primary platforms for disseminating influential findings, often publishing high‐impact studies and fostering international collaborations. This information is valuable for researchers aiming to identify potential collaborators, funding sources, and appropriate journals for future work in AI and CCTA.

Highly cited literature reflects the core advancements in this field. In 2016, the most cited article investigated the feasibility and accuracy of ML in predicting 5‐year all‐cause mortality (ACM) in patients undergoing CCTA, comparing its performance to existing clinical or CCTA metrics. The results showed that ML, combining clinical and CCTA data, significantly outperformed existing clinical or CCTA metrics alone in predicting 5‐year ACM [27]. Later in 2018, Al'Aref et al. explored how ML algorithms are being applied to enhance predictive modeling and diagnostic accuracy in non‐invasive imaging, such as coronary calcium scoring. This article also discussed the challenges associated with ML implementation in clinical practice, particularly regarding data quality and algorithm interpretability, underlining the transformative impact of ML in cardiac imaging [28]. The article ranked third in terms of citations is about the application of a novel AI‐derived radiomic signature, the Fat Radiomic Profile (FRP), in CCTA, which leverages perivascular adipose tissue characteristics captured in CCTA to improve cardiac risk prediction. This work underscores the integration of advanced AI techniques in radiomic profiling to capture complex tissue characteristics that standard imaging might miss, offering a personalized approach to risk assessment [8]. The articles ranked fourth and fifth are about the multifaceted applications of ML. Coenen et al. [29] evaluated an ML‐based approach to compute FFR from CCTA data, which improved diagnostic accuracy for coronary stenosis. Each of these studies highlights significant advancements in AI applications within CCTA, focusing on enhancing predictive capabilities, diagnostic accuracy, and clinical utility.

Keyword analysis provided insights into the evolving research focus within AI applications in CCTA. The keywords in this field are mainly present in three aspects: disease along with physiological foundations, AI technologies, and advanced imaging techniques. The keywords related to disease along with physiological foundations emerged early on and serve as the foundational bedrock of the research, reflecting the ongoing need to address CAD effectively. For instance, “coronary artery disease” (CAD) is a primary focus of CCTA, and AI techniques are increasingly being used to analyze CCTA images for early detection and management of CAD. “Atherosclerosis” is a key underlying process in CAD, and AI algorithms in CCTA are designed to identify and quantify “atherosclerotic plaques”, which can help predict the risk of heart attacks [30]. Additionally, the detection and characterization of “coronary stenosis” are crucial in CCTA, and AI techniques can enhance the accuracy and efficiency of this process, aiding in the diagnosis and treatment of CAD [31].

The keywords related to AI technologies reveal the development of AI technology and its multifaceted applications in CCTA. As fundamental in building AI models for CCTA analysis, “Machine learning” is a branch of AI that focuses on the development of algorithms that allow computers to learn from and make predictions based on data [32]. “Deep learning” is a subset of ML that involves training artificial neural networks to perform tasks such as image and speech recognition, enabling AI to detect and assess coronary artery abnormalities more accurately [6]. “Convolutional neural network” is a type of deep learning model that is widely used in CCTA image analysis for tasks such as plaque detection, segmentation, and classification because it excels in processing grid‐like data such as images [33]. “U‐Net” is a convolutional neural network architecture specifically designed for biomedical image segmentation. This model is widely used in CCTA image segmentation tasks, contributing to the precise identification and quantification of coronary artery structures and abnormalities [16].

Advanced imaging techniques such as “Fractional Flow Reserve” are measures of the pressure difference across coronary stenosis under maximal hyperemia, used to assess the hemodynamic significance of coronary artery stenosis [34]. AI techniques can enhance the accuracy and efficiency of FFR measurements in CCTA, aiding in the diagnosis and treatment planning of CAD [35, 36]. “Radiomics” involves the extraction of quantitative imaging features from medical images to provide a comprehensive representation of disease phenotypes. Radiomics can be applied to CCTA images to extract features that may be useful for predicting outcomes and guiding treatment decisions, enhancing the role of AI in CAD management [37]. Furthermore, AI techniques can help optimize CCTA protocols to reduce “radiation dose” while maintaining diagnostic accuracy, addressing concerns about radiation safety [38]. AI‐based analysis of CCTA images can aid in the planning and assessment of “transcatheter aortic valve implantation” (TAVI) procedures, ensuring optimal patient outcomes [39].

The burst analysis of keywords provided insight into the shifting research priorities over time. Before 2010, the keyword “follow‐up” experienced a prolonged burst period, reflecting the importance of longitudinal studies in understanding the progression of CAD. Between 2010 and 2020, the term “diagnostic accuracy” highlights the growing emphasis on AI‐enhanced precision in detecting CAD, particularly for subtle indicators that may be challenging to identify [40, 41]. The growing prominence of ML “prediction” tools from 2019 to 2022 aligns with broader efforts to integrate predictive algorithms into CCTA, facilitating earlier and more precise CAD detection and supporting timely interventions [42, 43]. ML enhances CCTA by advancing image analysis, plaque characterization, hemodynamic assessment, and predictive modeling. These advancements not only improve diagnostic accuracy and streamline workflows but also minimize the need for invasive diagnostics and enhance their utility when required [44, 45, 46].

The latest emerging keywords since 2021 are related to guidelines, indicating a focus on standardizing practices and ensuring quality in CCTA. The prominence of “SCCT Guidelines” and “Expert Consensus Document” reflects a growing emphasis on standardizing practices in CCTA, driven by advancements in AI that necessitate updated protocols to ensure diagnostic consistency. These guidelines aim to effectively incorporate AI within clinical workflows, enhancing accuracy and reproducibility across various healthcare settings. In line with this, the clinical practice statement by the American Society for Preventive Cardiology (ASPC) summarizes the current evidence and applications of cardiac CT in evaluating CAD. The statement explores emerging applications of CCTA, including those based on functional assessment using CT‐FFR, peri‐coronary inflammation, and AI. AI, in particular, can provide personalized risk assessment and guide targeted treatment, contributing to the role of CCTA as an essential tool in cardiovascular prevention, risk assessment, early diagnosis, and management. The document also notes potential areas for future investigation [47]. This shift towards standardization is crucial for translating AI advancements into clinical practice, ensuring that patients receive consistent and high‐quality care.

The evolution of research hotspots, from foundational topics to technological advancements and standardization, highlights the dynamic nature of the field. However, this evolution also reveals potential research bottlenecks. For instance, while AI algorithms have shown promise in improving diagnostic accuracy and prediction, there remains a need for more robust validation studies to confirm their clinical utility. Additionally, the integration of AI into clinical workflows and decision‐making processes remains a challenge, requiring further research and collaboration between clinicians and AI experts [48]. The potential direction suggested by keyword burst and citation patterns in AI in coronary CTA is likely to be driven by continued advancements in AI algorithms, increasing focus on validation and integration into clinical practice, and efforts to standardize practices and ensure quality care. By addressing these research bottlenecks and leveraging the insights provided by keyword analysis, the field can continue to evolve and improve patient outcomes.

The robustness of the present findings is supported by the sensitivity analysis. A narrower query focusing exclusively on established CCTA/CT‐FFR and AI terminology yielded highly concordant results across multiple bibliometric dimensions: the top 10 contributing countries (Jaccard index = 0.82), top 10 highly cited articles (overlap rate = 80%), and the core authors and journals were largely identical between the two search strategies. The peripheral ranking variations observed in the top 10 authors and journals (Jaccard index = 0.67 each) were attributable to the expanded temporal coverage of the narrow query rather than systematic divergence, as the leading 8 authors and 8 journals were shared. These results indicate that the broad query, combined with manual screening, captured the essential bibliometric structure of AI in CCTA research without introducing substantial bias from peripheral records.

## 5 Limitations

Despite its contributions, this study has limitations. First, we relied solely on a single database, potentially excluding relevant studies indexed elsewhere. Additionally, we did not include books or editorials, which might provide supplementary perspectives on AI applications in CCTA. The keyword analysis, while thorough, may overlook some newer or nuanced terms that could reflect evolving trends. The broad search strategy retrieved a proportion of non‐CCTA records (29% in a random 100‐article audit), which were removed through manual screening; although this cleaning step ensured dataset relevance, it introduced an element of subjective judgment. The sensitivity analysis using a narrower query was conducted at a later search date (May 2026) than the original retrieval (October 2024), introducing a temporal mismatch; nevertheless, the high concordance across core bibliometric dimensions supports the overall stability of the findings. Furthermore, the sensitivity analysis could not include annual publication trend correlation or keyword overlap due to the differing time windows, which limits the completeness of the cross‐validation. It should also be noted that bibliometric indicators reflect publication activity, citation structure, and thematic clustering rather than direct clinical significance or methodological quality of individual studies.

## 6 Conclusions

This study utilizes bibliometric analysis and network visualization to assess global research trends in AI applications for CCTA. Our findings show that research hotspots focus on AI‐enhanced diagnostic accuracy and guidelines for standardization, with ML prediction representing a bibliometrically prominent emerging theme with high citation burst intensity, suggesting intensifying research activity in this subdomain. This study provides a valuable resource for researchers interested in the field by highlighting key trends, influential authors, and collaborative institutions. The critical insights gained here have clinical implications, especially for integrating AI into routine CCTA practices, supporting improved diagnostic outcomes for CAD.

## Author Contributions

Conception and design: Shanshan Jiang. Administrative support: Yucong Zheng, Lu Ma. Data analysis and interpretation: Shanshan Jiang, Keqin Pan, Lu Ma. Manuscript writing: Shanshan Jiang. Final approval of manuscript: Shanshan Jiang, Yucong Zheng.

## Funding

The authors have nothing to report.

## Ethics Statement

The authors have nothing to report.

## Consent

The authors have nothing to report.

## Conflicts of Interest

The authors declare no conflicts of interest.

## Supporting information


**Table S1:** Top 50 articles with the highest cited counts. **Table S2:** Publication and citation profiles of leading countries. **Table S3:** Publication and citation profiles of high‐impact authors. **Table S4:** Bibliometric indicators of high‐impact journals. **Table S5:** Manual relevance audit of a random sample of 100 articles from the broad query dataset. **Table S6:** Sensitivity analysis comparing the broad query and narrow query datasets across multiple bibliometric dimensions.

## Data Availability

All data generated or analyzed during this study are included in this published article.
