# Supplementary material for: Artificial Intelligence in Coronary Computed Tomography Angiography: A Bibliometric Analysis of Trends and Themes
Source: Clin Cardiol. 2026 Aug 24;49(8):e70447. doi: 10.1002/clc.70447 (PMC13501894; doi:10.1002/clc.70447)
Supplement: Supplementary file 1 [file CLC-49-e70447-s001.docx]

**Supplementary Table 1.** Top 50 articles with the highest cited counts.

| **Paper** | **DOI** | **TC** | **TC per Year** | **Normalized TC** |
| --- | --- | --- | --- | --- |
| MOTWANI M, 2017, EUR HEART J | 10.1093/eurheartj/ehw188 | 472 | 59.00 | 5.41 |
| AL'AREF SJ, 2019, EUR HEART J | 10.1093/eurheartj/ehy404 | 269 | 44.83 | 4.50 |
| OIKONOMOU EK, 2019, EUR HEART J | 10.1093/eurheartj/ehz592 | 267 | 44.50 | 4.47 |
| COENEN A, 2018, CIRC-CARDIOVASC IMAG | 10.1161/CIRCIMAGING.117.007217 | 266 | 38.00 | 3.76 |
| ITU L, 2016, J APPL PHYSIOL | 10.1152/japplphysiol.00752.2015 | 265 | 29.44 | 1.99 |
| TATSUGAMI F, 2019, EUR RADIOL | 10.1007/s00330-019-06183-y | 169 | 28.17 | 2.83 |
| TESCHE C, 2018, RADIOLOGY | 10.1148/radiol.2018171291 | 162 | 23.14 | 2.29 |
| KANG E, 2019, MED PHYS | 10.1002/mp.13284 | 160 | 26.67 | 2.68 |
| GARCIA EV, 2007, J NUCL CARDIOL | 10.1016/j.nuclcard.2007.06.009 | 154 | 8.56 | 2.28 |
| ZREIK M, 2019, IEEE T MED IMAGING | 10.1109/TMI.2018.2883807 | 145 | 24.17 | 2.43 |
| AL'AREFILB SJ, 2020, EUR HEART J | 10.1093/eurheartj/ehz565 | 128 | 25.60 | 4.12 |
| ZREIK M, 2018, MED IMAGE ANAL | 10.1016/j.media.2017.11.008 | 124 | 17.71 | 1.75 |
| WOLTERINK JM, 2019, MED IMAGE ANAL | 10.1016/j.media.2018.10.005 | 120 | 20.00 | 2.01 |
| ZELEZNIK R, 2021, NAT COMMUN | 10.1038/s41467-021-20966-2 | 120 | 30.00 | 6.50 |
| VAN ROSENDAEL AR, 2018, J CARDIOVASC COMPUT | 10.1016/j.jcct.2018.04.011 | 119 | 17.00 | 1.68 |
| BENZ DC, 2020, J CARDIOVASC COMPUT | 10.1016/j.jcct.2020.01.002 | 109 | 21.80 | 3.51 |
| DEY D, 2018, EUR RADIOL | 10.1007/s00330-017-5223-z | 106 | 15.14 | 1.50 |
| LIN A, 2022, LANCET DIGIT HEALTH | NA | 103 | 34.33 | 11.08 |
| CHOI AD, 2021, J CARDIOVASC COMPUT | 10.1016/j.jcct.2021.05.004 | 94 | 23.50 | 5.09 |
| LIN A, 2020, JACC-CARDIOVASC IMAG | 10.1016/j.jcmg.2020.06.033 | 91 | 18.20 | 2.93 |
| RUEL M, 2014, J THORAC CARDIOV SUR | 10.1016/j.jtcvs.2013.09.016 | 90 | 8.18 | 2.14 |
| BIZOPOULOS P, 2019, IEEE REV BIOMED ENG | 10.1109/RBME.2018.2885714 | 86 | 14.33 | 1.44 |
| KOLOSSVARY M, 2019, RADIOLOGY | 10.1148/radiol.2019190407 | 85 | 14.17 | 1.42 |
| GAO ZF, 2020, NEURAL NETWORKS | 10.1016/j.neunet.2019.11.017 | 82 | 16.40 | 2.64 |
| DE VOS BD, 2017, IEEE T MED IMAGING | 10.1109/TMI.2017.2673121 | 81 | 10.13 | 0.93 |
| POSTON RS, 2008, ANN SURG | 10.1097/SLA.0b013e31818a15b5 | 76 | 4.47 | 1.00 |
| TESCHE C, 2020, JACC-CARDIOVASC IMAG | 10.1016/j.jcmg.2019.06.027 | 70 | 14.00 | 2.26 |
| LIN S, 2020, EUR HEART J | 10.1093/eurheartj/ehaa640 | 67 | 13.40 | 2.16 |
| NOOTHOUT JMH, 2020, IEEE T MED IMAGING | 10.1109/TMI.2020.3009002 | 66 | 13.20 | 2.13 |
| DOEBERITZ PLV, 2019, EUR RADIOL | 10.1007/s00330-018-5834-z | 63 | 10.50 | 1.05 |
| VAN ASSEN M, 2020, RADIOL MED | 10.1007/s11547-020-01277-w | 62 | 12.40 | 2.00 |
| VAN HAMERSVELT RW, 2019, EUR RADIOL | 10.1007/s00330-018-5822-3 | 60 | 10.00 | 1.00 |
| MUSCOGIURI G, 2020, ATHEROSCLEROSIS | 10.1016/j.atherosclerosis.2019.12.001 | 56 | 11.20 | 1.80 |
| SENGUPTA PP, 2018, J AM COLL CARDIOL | 10.1016/j.jacc.2018.02.024 | 54 | 7.71 | 0.76 |
| KONG B, 2020, COMPUT MED IMAG GRAP | 10.1016/j.compmedimag.2019.101688 | 52 | 10.40 | 1.68 |
| YU MM, 2020, EUR RADIOL | 10.1007/s00330-019-06400-8 | 52 | 10.40 | 1.68 |
| BERNARD A, 2021, QUANT IMAG MED SURG | 10.21037/qims-20-626 | 52 | 13.00 | 2.82 |
| LI YH, 2019, RADIOLOGY | 10.1148/radiol.2019190098 | 52 | 8.67 | 0.87 |
| ADAMS C, 2014, EUR J CARDIO-THORAC | 10.1093/ejcts/ezt390 | 52 | 4.73 | 1.24 |
| WANG ZQ, 2019, J GERIATR CARDIOL | 10.11909/j.issn.1671-5411.2019.01.010 | 50 | 8.33 | 0.84 |
| SHEN Y, 2019, IEEE ACCESS | 10.1109/ACCESS.2019.2908039 | 49 | 8.17 | 0.82 |
| SLART RHJA, 2021, EUR J NUCL MED MOL I | 10.1007/s00259-021-05341-z | 49 | 12.25 | 2.66 |
| DEY D, 2015, CIRC-CARDIOVASC IMAG | 10.1161/CIRCIMAGING.115.003255 | 49 | 4.90 | 1.42 |
| GRIFFIN WF, 2023, JACC-CARDIOVASC IMAG | 10.1016/j.jcmg.2021.10.020 | 48 | 24.00 | 9.71 |
| CURRIE ME, 2012, ANN THORAC SURG | 10.1016/j.athoracsur.2011.11.031 | 48 | 3.69 | 1.00 |
| HAN D, 2020, COMPUT METH PROG BIO | 10.1016/j.cmpb.2020.105651 | 46 | 9.20 | 1.48 |
| SANDHU AT, 2023, CIRCULATION | 10.1161/CIRCULATIONAHA.122.062746 | 46 | 23.00 | 9.30 |
| HONG JH, 2020, KOREAN J RADIOL | 10.3348/kjr.2020.0020 | 45 | 9.00 | 1.45 |
| JOHNSON KM, 2019, RADIOLOGY | 10.1148/radiol.2019182061 | 45 | 7.50 | 0.75 |
| YANG S, 2021, JACC-CARDIOVASC IMAG | 10.1016/j.jcmg.2020.08.025 | 44 | 11.00 | 2.38 |

Notes: TC: Total Citations.

**Supplementary Table 2.** Publication and citation profiles of leading countries.

| **Country** | **Articles** | **Freq** | **SCP** | **MCP** | **MCP-Ratio** | **TP** | **TP-rank** | **TC** | **TC-rank** | **Average Citations** |
| --- | --- | --- | --- | --- | --- | --- | --- | --- | --- | --- |
| CHINA | 193 | 38.68 | 149 | 44 | 22.80 | 735 | 1 | 1791 | 2 | 9.3 |
| USA | 110 | 22.04 | 30 | 80 | 72.73 | 722 | 2 | 3651 | 1 | 33.2 |
| KOREA | 30 | 6.01 | 27 | 3 | 10.00 | 256 | 3 | 480 | 4 | 16 |
| NETHERLANDS | 30 | 6.01 | 12 | 18 | 60.00 | 188 | 5 | 1104 | 3 | 36.8 |
| GERMANY | 26 | 5.21 | 15 | 11 | 42.31 | 233 | 4 | 230 | 7 | 8.8 |
| JAPAN | 21 | 4.21 | 13 | 8 | 38.10 | 158 | 7 | 344 | 6 | 16.4 |
| ITALY | 14 | 2.81 | 8 | 6 | 42.86 | 160 | 6 | 222 | 8 | 15.9 |
| UNITED KINGDOM | 14 | 2.81 | 5 | 9 | 64.29 | 118 | 8 | 378 | 5 | 27 |
| CANADA | 5 | 1.00 | 1 | 4 | 80.00 | 45 | 9 | 206 | 9 | 41.2 |
| FINLAND | 5 | 1.00 | 1 | 4 | 80.00 | 24 | 14 | 3 | 29 | 0.6 |
| FRANCE | 5 | 1.00 | 4 | 1 | 20.00 | 29 | 12 | 85 | 12 | 17 |
| POLAND | 5 | 1.00 | 3 | 2 | 40.00 | 26 | 13 | 57 | 14 | 11.4 |
| ISRAEL | 4 | 0.80 | 3 | 1 | 25.00 | 10 | 26 | 46 | 15 | 11.5 |
| SWEDEN | 4 | 0.80 | 1 | 3 | 75.00 | 40 | 10 | 7 | 26 | 1.8 |
| SWITZERLAND | 4 | 0.80 | 2 | 2 | 50.00 | 34 | 11 | 155 | 10 | 38.8 |
| GREECE | 3 | 0.60 | 1 | 2 | 66.67 | 7 | 29 | 93 | 11 | 31 |
| INDIA | 3 | 0.60 | 2 | 1 | 33.33 | 21 | 17 | 19 | 19 | 6.3 |
| IRAN | 3 | 0.60 | 2 | 1 | 33.33 | 12 | 23 | 7 | 25 | 2.3 |
| AUSTRALIA | 2 | 0.40 | 2 | 0 | 0.00 | 20 | 18 | 26 | 16 | 13 |
| CROATIA | 2 | 0.40 | 2 | 0 | 0.00 | 5 | 31 | 7 | 24 | 3.5 |

Note(s): Articles: Publications of Corresponding Authors only. Freq: Frequence of Total Publications. MCP-Ratio: Proportion of Multiple Country Publications. TP: Total Publications. TP-rank: Rank of Total Publications. TC: Total Citations. TC-rank: Rank of Total Citations. Average Citations: The average number of citations per publication.

**Supplementary Table 3.** Publication and citation profiles of high-impact authors.

| **Authors** | **H-index** | **G-index** | **M-index** | **PY-start** | **TP** | **TP-rank** | **TC** | **TC-rank** |
| --- | --- | --- | --- | --- | --- | --- | --- | --- |
| SCHOEPF U. JOSEPH | 16 | 36 | 2 | 2017 | 36 | 1 | 271 | 1 |
| MIN JAMES K. | 15 | 31 | 1.5 | 2015 | 31 | 2 | 146 | 10 |
| CHANG HYUK-JAE | 14 | 21 | 1.75 | 2017 | 21 | 5 | 154 | 9 |
| PONTONE GIANLUCA | 14 | 27 | 1.75 | 2017 | 27 | 3 | 165 | 7 |
| BERMAN DANIEL S. | 13 | 17 | 1.3 | 2015 | 17 | 9 | 142 | 11 |
| TESCHE CHRISTIAN | 13 | 22 | 1.625 | 2017 | 22 | 4 | 199 | 2 |
| VARGA-SZEMES AKOS | 12 | 15 | 1.5 | 2017 | 15 | 11 | 70 | 58 |
| ACHENBACH STEPHAN | 11 | 13 | 1.1 | 2015 | 13 | 16 | 135 | 12 |
| ANDREINI DANIELE | 11 | 20 | 1.375 | 2017 | 20 | 7 | 156 | 8 |
| DE CECCO CARLO N. | 11 | 14 | 1.375 | 2017 | 14 | 13 | 135 | 13 |
| DEY DAMINI | 11 | 13 | 1.1 | 2015 | 13 | 17 | 96 | 28 |
| MARQUES HUGO | 11 | 16 | 1.375 | 2017 | 16 | 10 | 165 | 6 |
| SLOMKA PIOTR J. | 11 | 11 | 1.1 | 2015 | 11 | 27 | 111 | 21 |
| ISGUM IVANA | 10 | 14 | 1.25 | 2017 | 14 | 14 | 114 | 16 |
| SHAW LESLEE J. | 10 | 13 | 1.25 | 2017 | 13 | 19 | 114 | 18 |
| GRANSAR HEIDI | 9 | 9 | 0.9 | 2015 | 9 | 42 | 117 | 15 |
| HAN DONGHEE | 9 | 10 | 1.285714 | 2018 | 10 | 31 | 32 | 145 |
| LU GUANG MING | 9 | 10 | 1.5 | 2019 | 10 | 33 | 50 | 80 |
| ZHANG LONG JIANG | 9 | 13 | 1.5 | 2019 | 13 | 20 | 53 | 74 |
| ZHOU CHANG SHENG | 9 | 10 | 1.5 | 2019 | 10 | 36 | 50 | 81 |

Note(s): H-index: The h-index measures both the productivity and citation impact of the publications. G-index: The g-index gives more weight to highly cited articles. M-index: The m-index is the h-index divided by the number of years since the first published paper. PY-start: Publication Year Start, indicating the year the journal started publication.TP: Total Publications. TP-rank: Rank of Total Publications. TC: Total Citations. TC-rank: Rank of Total Citations. Average Citations: The average number of citations per publication.

**Supplementary Table 4.** Bibliometric indicators of high-impact journals.

| **Journal** | **H-index** | **G-index** | **M-index** | **IF** | **JCR-Quartile** | **PY-start** | **TP** | **TP-rank** | **TC** | **TC-rank** |
| --- | --- | --- | --- | --- | --- | --- | --- | --- | --- | --- |
| EUROPEAN RADIOLOGY | 16 | 28 | 2.29 | 4.7 | Q1 | 2018 | 35 | 1 | 609 | 5 |
| JACC-CARDIOVASCULAR IMAGING | 12 | 14 | 2.40 | 12.8 | Q1 | 2020 | 14 | 7 | 880 | 2 |
| RADIOLOGY | 9 | 10 | 1.29 | 12.1 | Q1 | 2018 | 10 | 11 | 599 | 6 |
| JOURNAL OF CARDIOVASCULAR COMPUTED TOMOGRAPHY | 8 | 15 | 1.00 | 5.5 | Q1 | 2017 | 15 | 4 | 862 | 3 |
| MEDICAL PHYSICS | 8 | 15 | 0.73 | 3.2 | Q1 | 2014 | 15 | 5 | 215 | 15 |
| EUROPEAN JOURNAL OF RADIOLOGY | 7 | 12 | 1.17 | 3.2 | Q1 | 2019 | 12 | 9 | 219 | 14 |
| ACADEMIC RADIOLOGY | 6 | 8 | 1.20 | 3.8 | Q1 | 2020 | 10 | 10 | 127 | 24 |
| EUROPEAN HEART JOURNAL | 6 | 7 | 0.75 | 37.6 | Q1 | 2017 | 7 | 18 | 660 | 4 |
| EUROPEAN HEART JOURNAL-CARDIOVASCULAR IMAGING | 6 | 11 | 1.20 | 6.7 | Q1 | 2020 | 12 | 8 | 264 | 12 |
| IEEE TRANSACTIONS ON MEDICAL IMAGING | 6 | 8 | 0.75 | 8.9 | Q1 | 2017 | 8 | 15 | 343 | 9 |
| DIAGNOSTICS | 5 | 7 | 1.25 | 3 | Q1 | 2021 | 14 | 6 | 37 | 72 |
| FRONTIERS IN CARDIOVASCULAR MEDICINE | 5 | 9 | 1.25 | 2.8 | Q2 | 2021 | 20 | 2 | 103 | 29 |
| INTERNATIONAL JOURNAL OF CARDIOLOGY | 5 | 7 | 0.71 | 3.2 | Q2 | 2018 | 7 | 19 | 119 | 26 |
| MEDICAL IMAGE ANALYSIS | 5 | 6 | 0.50 | 10.7 | Q1 | 2015 | 6 | 29 | 341 | 10 |
| AMERICAN JOURNAL OF CARDIOLOGY | 4 | 8 | 0.50 | 2.3 | Q2 | 2017 | 8 | 12 | 187 | 17 |
| AMERICAN JOURNAL OF ROENTGENOLOGY | 4 | 6 | 0.67 | 4.7 | Q1 | 2019 | 6 | 23 | 154 | 21 |
| BMC MEDICAL IMAGING | 4 | 5 | 1.00 | 2.9 | Q2 | 2021 | 6 | 24 | 36 | 75 |
| CIRCULATION-CARDIOVASCULAR IMAGING | 4 | 6 | 0.40 | 6.5 | Q1 | 2015 | 6 | 25 | 252 | 13 |
| COMPUTERIZED MEDICAL IMAGING AND GRAPHICS | 4 | 7 | 0.57 | 5.4 | Q1 | 2018 | 7 | 17 | 80 | 38 |
| IEEE ACCESS | 4 | 4 | 0.67 | 3.4 | Q2 | 2019 | 4 | 37 | 85 | 36 |

Note(s): H-index: The h-index of the journal, which measures both the productivity and citation impact of the publications. G-index: The g-index of journal, which gives more weight to highly cited articles. M-index: The m-index of journal, which is the h-index divided by the number of years since the first published paper. IF: Impact Factor, indicating the average number of citations to recent articles published in the journal. JCR-Quartile: The quartile ranking of the journal in the Journal Citation Reports, indicating the journal's ranking relative to others in the same field (Q1: top 25%, Q2: 25%-50%, Q3: 50%-75%, Q4: bottom 25%). PY-start: Publication Year Start, indicating the year the journal started publication. TP: Total Publications. TP-rank: Rank of Total Publications. TC: Total Citations. TC-rank: Rank of Total Citations.

**Supplementary Table S5.** Manual relevance audit of a random sample of 100 articles from the broad query dataset (set.seed = 20260422).

| **No.** | **WoS_ID** | **Year** | **Title** | **Relevance** | **Exclusion Rationale** |
| --- | --- | --- | --- | --- | --- |
| 1 | WOS:000527012600016 | 2020 | Machine Learning Framework to Identify Individuals at Risk o... | Included | -- |
| 2 | WOS:000720333000001 | 2021 | Diagnostic Accuracy and Generalizability of a Deep Learning-... | Included | -- |
| 3 | WOS:001103396400012 | 2023 | Deep Learning-Based Motion Correction in Projection Domain f... | Included | -- |
| 4 | WOS:000762570700001 | 2021 | Recent Trends in Artificial Intelligence-Assisted Coronary A... | Included | -- |
| 5 | WOS:001228899100003 | 2024 | Predicting major adverse cardiovascular events within 3 year... | Included | -- |
| 6 | WOS:001316620600001 | 2024 | Predictors of discordance between CT-derived fractional flow... | Included | -- |
| 7 | WOS:001070965800001 | 2023 | Relation of Gender to Atherosclerotic Plaque Characteristics... | Included | -- |
| 8 | WOS:000516985700001 | 2020 | Automated left ventricular myocardium segmentation using 3D ... | Included | -- |
| 9 | WOS:001006579600001 | 2023 | Assessment of Image Quality of Coronary CT Angiography Using... | Included | -- |
| 10 | WOS:000759110000001 | 2022 | Image restoration of motion artifacts in cardiac arteries an... | Included | -- |
| 11 | WOS:000769348000002 | 2022 | Automatic coronary plaque detection, classification, and ste... | Included | -- |
| 12 | WOS:000757369300001 | 2022 | One-year outcomes of CCTA alone versus machine learning-base... | Included | -- |
| 13 | WOS:000566349400004 | 2021 | Prediction of revascularization by coronary CT angiography u... | Included | -- |
| 14 | WOS:001205461900003 | 2024 | A comparative analysis of deep learning-based location-adapt... | Included | -- |
| 15 | WOS:000656660500006 | 2021 | Radiomic features of plaques derived from coronary CT angiog... | Included | -- |
| 16 | WOS:000413887400006 | 2017 | Coronary Computed Tomographic Angiography-Derived Fractional... | Included | -- |
| 17 | WOS:000971282600001 | 2023 | Validation of the commercial coronary computed tomographic a... | Included | -- |
| 18 | WOS:000295255700017 | 2011 | Early and midterm results of totally endoscopic coronary art... | Excluded | CCTA used solely as post-surgical follow-up tool for graft patency assessment, not as the subject of AI application |
| 19 | WOS:000463157200021 | 2019 | Deep learning analysis of left ventricular myocardium in CT ... | Included | -- |
| 20 | WOS:000632508800015 | 2021 | CT Angiographic and Plaque Predictors of Functionally Signif... | Included | -- |
| 21 | WOS:001316297800001 | 2024 | Ruptured left sinus of Valsalva aneurysm into the left atriu... | Excluded | Veterinary case report (Valsalva sinus aneurysm); not AI-related and not CCTA |
| 22 | WOS:000671732000025 | 2021 | A radiomic approach to predict myocardial fibrosis on corona... | Included | -- |
| 23 | WOS:000537283500043 | 2020 | Automatic segmentation of multiple cardiovascular structures... | Included | -- |
| 24 | WOS:000418623600001 | 2017 | Coronary Computed Tomographic Angiography-Derived Fractional... | Included | -- |
| 25 | WOS:000747710200003 | 2022 | Artificial Intelligence Segmentation Model-Based Computed To... | Excluded | Non-coronary CTA: congenital aortic coarctation CTA |
| 26 | WOS:000755805700001 | 2022 | Risk predicting for acute coronary syndrome based on machine... | Included | -- |
| 27 | WOS:001165219500001 | 2024 | Effect of deep learning image reconstruction with high-defin... | Included | -- |
| 28 | WOS:000748345400004 | 2022 | In Vitro Study of the Precision and Accuracy of Measurement ... | Included | -- |
| 29 | WOS:000585936200013 | 2020 | Myocardial Infarction Associates With a Distinct Pericoronar... | Included | -- |
| 30 | WOS:000941885500002 | 2023 | Coronary computed tomography angiography imaging features co... | Included | -- |
| 31 | WOS:000856648600001 | 2022 | Development and validation of a machine learned algorithm to... | Excluded | Non-CCTA imaging: ML algorithm based on facial photographs and electrical signals |
| 32 | WOS:000541250900014 | 2020 | Impact of machine-learning CT-derived fractional flow reserv... | Included | -- |
| 33 | WOS:000565532900003 | 2020 | Supervised Filter Learning for Coronary Artery Vesselness En... | Included | -- |
| 34 | WOS:000748345400007 | 2022 | Assessment of Image Quality of Coronary Computed Tomography ... | Included | -- |
| 35 | WOS:001117190400001 | 2023 | Human AI Teaming for Coronary CT Angiography Assessment: Imp... | Included | -- |
| 36 | WOS:000465338700001 | 2019 | Coronary Arteries Segmentation Based on 3D FCN With Attentio... | Included | -- |
| 37 | WOS:000978842200004 | 2023 | On-Site Computed Tomography-Derived Fractional Flow Reserve ... | Included | -- |
| 38 | WOS:001308601400001 | 2024 | Evaluating coronary arteries and predicting MACEs using CCTA... | Included | -- |
| 39 | WOS:001205105500003 | 2024 | Lesion Classification of Coronary Artery CTA Images Based on... | Included | -- |
| 40 | WOS:000569569800001 | 2020 | The sub-millisievert era in CTCA: the technical basis of the... | Excluded | Non-AI study: review of radiation dose reduction and scanning techniques without AI/ML component |
| 41 | WOS:000640864200001 | 2021 | Position paper of the EACVI and EANM on artificial intellige... | Included | -- |
| 42 | WOS:000448657700001 | 2018 | An Adaptive Seismocardiography (SCG)-ECG Multimodal Framewor... | Excluded | Non-CCTA imaging: SCG-ECG gating technology; AI applied to gating signal processing rather than CCTA analysis |
| 43 | WOS:000746484000009 | 2022 | Prognostic value of epicardial adipose tissue volume in comb... | Included | -- |
| 44 | WOS:001128678500001 | 2023 | Combining generative modelling and semi-supervised domain ad... | Excluded | Non-CCTA imaging: cardiac MRI angiography segmentation |
| 45 | WOS:000862400100014 | 2022 | CAR-Net: A Deep Learning-Based Deformation Model for 3D/2D C... | Included | -- |
| 46 | WOS:001097318300001 | 2024 | Ori-Net: Orientation-guided Neural Network for Automated Cor... | Included | -- |
| 47 | WOS:001097005400002 | 2023 | A deep learning-based automated algorithm for labeling coron... | Included | -- |
| 48 | WOS:000714977600005 | 2021 | CT Evaluation by Artificial Intelligence for Atherosclerosis... | Included | -- |
| 49 | WOS:000575788600019 | 2020 | Validation of deep-learning image reconstruction for coronar... | Included | -- |
| 50 | WOS:000730511500052 | 2021 | Prognostic value of pericoronary inflammation and unsupervis... | Included | -- |
| 51 | WOS:000903374100001 | 2023 | Improved image quality in transcatheter aortic valve implant... | Excluded | Non-coronary CTA: TAVI planning CT, not coronary CTA |
| 52 | WOS:000463157200024 | 2019 | Coronary CT angiography-derived plaque quantification with a... | Included | -- |
| 53 | WOS:000874004800001 | 2022 | Machine-learning-derived radiomics signature of pericoronary... | Included | -- |
| 54 | WOS:000943493400008 | 2023 | Incidental Coronary Artery Calcium: Opportunistic Screening ... | Excluded | Non-coronary CTA: non-gated chest CT for opportunistic CAC screening, not CCTA protocol |
| 55 | WOS:000248573600002 | 2007 | The increasing role of quantification in clinical nuclear ca... | Excluded | Non-CCTA imaging: nuclear medicine SPECT/PET quantification; not AI-related |
| 56 | WOS:000877585500002 | 2022 | Regional left ventricle scar detection from routine cardiac ... | Included | -- |
| 57 | WOS:000606543900012 | 2020 | Feasibility of using deep learning to detect coronary artery... | Excluded | Non-CCTA imaging: DL algorithm based on facial photographs for CAD detection |
| 58 | WOS:000984284300060 | 2023 | How scan parameter choice affects deep learning-based corona... | Included | -- |
| 59 | WOS:000656473400001 | 2021 | Vessel segmentation from volumetric images: a multi-scale do... | Included | -- |
| 60 | WOS:000719561600006 | 2021 | Examinee-Examiner Network: Weakly Supervised Accurate Corona... | Included | -- |
| 61 | WOS:000751120500008 | 2021 | Deep Learning Algorithm Predicts Angiographic Coronary Arter... | Excluded | Non-CCTA imaging: 12-lead ECG-based DL for CAD prediction |
| 62 | WOS:000468361000001 | 2019 | Accurate Segmentation of Heart Volume in CTA With Landmark-B... | Included | -- |
| 63 | WOS:000991196800003 | 2023 | Feasibility of accelerated non-contrast-enhanced whole-heart... | Excluded | Non-CCTA imaging: coronary MR angiography |
| 64 | WOS:001309800300001 | 2024 | Correlation between left atrial appendage morphology based o... | Excluded | Non-coronary cardiac structure: left atrial appendage morphology in atrial fibrillation; not AI-related |
| 65 | WOS:001133375600010 | 2023 | A novel deep learning model for a computed tomography diagno... | Included | -- |
| 66 | WOS:000670960600004 | 2021 | Eleven-year outcomes of U-clips in totally robotic coronary ... | Excluded | Unrelated topic: robotic CABG surgery outcomes; not AI in CCTA |
| 67 | WOS:000890955000001 | 2022 | Real-time 3D echocardiographic transilluminated imaging comb... | Excluded | Non-CCTA imaging: 3D echocardiography with AI for left atrial appendage measurement |
| 68 | WOS:000940080800001 | 2023 | Deep Learning-Based Acceleration of Compressed Sensing for N... | Excluded | Non-CCTA imaging: coronary MR angiography |
| 69 | WOS:000975331600001 | 2023 | Identification of ischemia-causing lesions using coronary pl... | Included | -- |
| 70 | WOS:001102105400001 | 2023 | Coronary artery assessment on pre transcatheter aortic valve... | Excluded | Non-coronary CTA: TAVI pre-procedural CT for coronary assessment, not standard CCTA protocol |
| 71 | WOS:001262939800001 | 2024 | Coronary artery disease evaluation during transcatheter aort... | Included | -- |
| 72 | WOS:001272861800001 | 2024 | Lipoprotein(a) and Long-Term Plaque Progression, Low-Density... | Included | -- |
| 73 | WOS:000358838400002 | 2015 | Quantitative Evaluation of the Substantially Variable Morpho... | Excluded | Non-coronary cardiac structure: left atrial appendage morphology; not AI-related |
| 74 | WOS:000451827800010 | 2018 | Machine learning assessment of myocardial ischemia using ang... | Included | -- |
| 75 | WOS:001224101300001 | 2024 | The Role of Artificial Intelligence in Cardiac Imaging | Included | -- |
| 76 | WOS:000788104800004 | 2022 | Machine Learning in Cardiovascular Imaging | Included | -- |
| 77 | WOS:001141436600001 | 2023 | Real-world approach to comprehensive artificial intelligence... | Included | -- |
| 78 | WOS:001050365500004 | 2023 | Artificial intelligence in estimating fractional flow reserv... | Included | -- |
| 79 | WOS:000532214700024 | 2020 | Deep Learning Analysis of Coronary Arteries in Cardiac CT An... | Included | -- |
| 80 | WOS:000860769700001 | 2022 | Learning coronary artery calcium scoring in coronary CTA fro... | Included | -- |
| 81 | WOS:000819692500010 | 2022 | Vessel-GAN: Angiographic reconstructions from myocardial CT ... | Excluded | Non-CCTA imaging: myocardial CT perfusion-based angiographic reconstruction |
| 82 | WOS:000894249200003 | 2022 | Deep learning-based prediction of coronary artery stenosis r... | Included | -- |
| 83 | WOS:001311006600001 | 2024 | Interrater Variability of ML-Based CT-FFR in Patients withou... | Included | -- |
| 84 | WOS:001050203700001 | 2023 | Calculation of left ventricular ejection fraction using an 8... | Included | -- |
| 85 | WOS:000742856700016 | 2022 | Predicting haemodynamic significance of coronary stenosis wi... | Included | -- |
| 86 | WOS:000404981000009 | 2017 | ConvNet-Based Localization of Anatomical Structures in 3-D M... | Included | -- |
| 87 | WOS:000878640700003 | 2023 | Two-stage training strategy combined with neural network for... | Excluded | Non-coronary CTA: internal mammary artery graft segmentation |
| 88 | WOS:000734670200001 | 2022 | Automating fractional flow reserve (FFR) calculation from CT... | Included | -- |
| 89 | WOS:001065367900001 | 2023 | Segmentation of cardiac tissues and organs for CCTA images b... | Included | -- |
| 90 | WOS:000804483900001 | 2022 | Temporal assessment of lesion morphology on radiological ima... | Included | -- |
| 91 | WOS:001049683100001 | 2023 | A knowledge graph-based analytical model for mining clinical... | Excluded | Non-CCTA imaging: drug-loaded stress echocardiography |
| 92 | WOS:000544052200001 | 2020 | A Unique Signature of Cardiac-Induced Cranial Forces During ... | Excluded | Unrelated topic: cranial accelerometer for stroke detection |
| 93 | WOS:000795339200001 | 2022 | Autostereoscopic 3D Display System for 3D Medical Images | Excluded | Non-AI study: 3D display system without AI diagnostic/analytic component |
| 94 | WOS:001099849300001 | 2023 | Stress-only dynamic computed tomography perfusion protocol (... | Excluded | Non-CCTA imaging: CT perfusion (CTP) protocol |
| 95 | WOS:000702638800009 | 2021 | Few-Shot Learning by a Cascaded Framework With Shape-Constra... | Included | -- |
| 96 | WOS:000978761800013 | 2023 | Deep Learning Analysis of Chest Radiographs to Triage Patien... | Excluded | Non-CCTA imaging: chest radiograph-based DL |
| 97 | WOS:000665290100001 | 2021 | Computed Tomography Imaging in Simulated Ongoing Cardiopulmo... | Excluded | Unrelated topic: CT imaging during cardiopulmonary resuscitation; not AI-related |
| 98 | WOS:000934111100008 | 2022 | Automated analysis of fibrous cap in intravascular optical c... | Excluded | Non-CCTA imaging: intravascular OCT fibrous cap analysis |
| 99 | WOS:000852745600001 | 2022 | Segmentation and volume quantification of epicardial adipose... | Included | -- |
| 100 | WOS:000740923700002 | 2022 | Transcarotid access for remote robotic endovascular neuroint... | Excluded | Unrelated topic: transcarotid neurointerventional procedure |

Note(s): A random sample of 100 articles was drawn from the broad query retrieval (set.seed = 20260422) for manual relevance screening. Of the 100 articles, 71 (71%) were confirmed as relevant and 29 (29%) were excluded. Exclusion categories: non-CCTA imaging modality (cardiac MRI, IVOCT, echocardiography, chest radiograph, CT perfusion, facial photograph, ECG-based AI, coronary MR angiography), n = 14; non-coronary CTA (TAVI-CT, aortic coarctation CTA, internal mammary artery graft, non-gated chest CT), n = 5; completely unrelated topics (surgery, stroke, veterinary medicine, neurointerventional procedure), n = 5; non-AI studies (radiation dose technique, 3D display system), n = 2; non-coronary cardiac structures (left atrial appendage), n = 2; CCTA as post-surgical follow-up tool only, n = 1.

**Supplementary Table S6.** Sensitivity analysis comparing the broad query dataset (n = 499, retrieved October 15, 2024) and the narrow query dataset (retrieved May 22, 2026) across multiple bibliometric dimensions.

| **Dimension** | **Broad Query Top 10** | **Narrow Query Top 10** | **Shared Entries** | **Metric** | **Value** | **Threshold** | **Conclusion** |
| --- | --- | --- | --- | --- | --- | --- | --- |
| Top 10 Countries | China, USA, Korea, Netherlands, Germany, Japan, Italy, United Kingdom, Canada, Finland | China, USA, Netherlands, Germany, Korea, Japan, Italy, United Kingdom, Finland, India | China, USA, Netherlands, Germany, Korea, Japan, Italy, United Kingdom, Finland (9) | Jaccard Index | 0.82 (9/11) | > 0.80 | Concordant |
| Top 10 Authors (by h-index) | Schoepf UJ, Min JK, Chang HJ, Pontone G, Berman DS, Tesche C, Varga-Szemes A, Achenbach S, Andreini D, De Cecco CN | Schoepf UJ, Min JK, Pontone G, Andreini D, Tesche C, Achenbach S, Berman DS, Chang HJ, Choi AD, Zhang LJ | Schoepf UJ, Min JK, Pontone G, Andreini D, Tesche C, Achenbach S, Berman DS, Chang HJ (8) | Jaccard Index | 0.67 (8/12) | > 0.80 | Borderline |
| Top 10 Journals (by h-index) | Eur Radiol, JACC-Cardiovasc Imaging, Radiology, J Cardiovasc Comput Tomogr, Med Phys, Eur J Radiol, Acad Radiol, Eur Heart J, Eur Heart J-Cardiovasc Imaging, IEEE Trans Med Imaging | Eur Radiol, JACC-Cardiovasc Imaging, J Cardiovasc Comput Tomogr, Eur Heart J-Cardiovasc Imaging, Radiology, Med Phys, Acad Radiol, Circ-Cardiovasc Imaging, Eur J Radiol, BMC Med Imaging | Eur Radiol, JACC-Cardiovasc Imaging, Radiology, J Cardiovasc Comput Tomogr, Med Phys, Eur J Radiol, Acad Radiol, Eur Heart J-Cardiovasc Imaging (8) | Jaccard Index | 0.67 (8/12) | > 0.80 | Borderline |
| Top 10 Highly Cited Articles | Motwani M (2017), Al'Aref SJ (2019), Oikonomou EK (2019), Coenen A (2018), Itu L (2016), Tatsugami F (2019), Tesche C (2018), Kang E (2019), Garcia EV (2007), Zreik M (2019) | Motwani M (2017), Oikonomou EK (2019), Al'Aref SJ (2019), Coenen A (2018), Itu L (2016), Lin A (2022), Tatsugami F (2019), Zreik M (2019), Chan K (2024), Kang E (2019) | Motwani M (2017), Oikonomou EK (2019), Al'Aref SJ (2019), Coenen A (2018), Itu L (2016), Tatsugami F (2019), Zreik M (2019), Kang E (2019) (8) | Overlap Rate | 0.80 (8/10) | ≥ 8/10 | Concordant |
| Narrow-query-unique Highly Cited Articles not covered by Broad Query Top 50 | -- | Lin A (2022): covered in Broad Top 50 (Rank 18); Chan K (2024): not covered | -- | Uncovered count | 1 | < 3 | Concordant |

Note(s): The broad query dataset comprised 499 articles retrieved on October 15, 2024. The narrow query was executed on May 22, 2026, using a more restrictive search string focused on core CCTA/CT-FFR terminology and established AI method terms; the wider temporal window represents a conservative validation design, as concordance despite expanded coverage further supports the robustness of the original dataset. Jaccard Index = |A∩B| / |A∪B|, where A and B are the respective top-N lists. Overlap Rate = number of shared articles / 10. Predefined concordance thresholds: Jaccard > 0.80 for countries, authors, and journals; overlap ≥ 8/10 for highly cited articles; < 3 uncovered articles for narrow-query-unique detection. For the Top 10 Authors and Top 10 Journals dimensions, although the Jaccard indices (0.67) fell below the 0.80 threshold, the core 8 entries were identical between the two queries; peripheral ranking differences were attributable to the expanded temporal coverage and minor variations in retrieval scope rather than systematic divergence.
